# Antibodies against Rift Valley Fever Virus in Cattle, Mozambique

**DOI:** 10.3201/eid1907.130332

**Published:** 2013-07

**Authors:** Nina Lagerqvist, Belisário Moiane, Lourenço Mapaco, José Fafetine, Sirkka Vene, Kerstin I. Falk

**Affiliations:** Karolinska Institutet, Stockholm, Sweden (N. Lagerqvist, B. Moiane, K.I. Falk);; Swedish Institute for Communicable Disease Control, Solna, Sweden (N. Lagerqvist, S. Vene, K.I. Falk);; Universidade Eduardo Mondlane, Maputo, Mozambique (B. Moiane, J. Fafetine);; Direcção de Ciências Animais, Maputo (L. Mapaco)

**Keywords:** Rift Valley fever virus, FVFV, viruses, bovine, cattle, antibodies, seroprevalence, Mapoto Province, Mozambique

**To the Editor:** During the past 2 decades, several countries in Africa and the Arabian Peninsula, to which Rift Valley fever virus (RVFV) is endemic, have reported outbreaks of Rift Valley fever in humans and livestock. The first evidence of RVFV in Mozambique was documented as early as the 1960s (*1*). Endemicity was subsequently confirmed in the 1980s by a prevalence study that identified virus-specific antibodies in 2% of pregnant women (*2*) and in the 1990s by serosurveillance in Zambezia Province, which showed that cattle had been infected with RVFV (*3*).

Apart from those observations, the RVFV situation in Mozambique is still poorly understood. We recently found an unexpectedly high level of RVFV activity in cattle in Namaacha District in Maputo Province (*4*), a region where there had been no recorded evidence of the virus since 1969 (*1*). We conducted a cross-sectional study in which serum samples were collected throughout Maputo Province during 2010–2011 to ascertain whether any RVFV circulation had remained undetected among bovids.

The study was approved by the Mozambican Board of Agriculture. Animals investigated were of mixed breed, had been present in their respective localities since birth, were >6 months of age, and had not been vaccinated against RVFV. Samples were analyzed by using a plaque-reduction neutralization test (*4*), and RVFV seropositivity was defined as 80% reduction of virus infectivity at a serum dilution of 1:40.

A total of 404 serum samples were analyzed, and 149 were positive for RVFV-neutralizing antibodies. This finding represents an overall seroprevalence of 36.9% (95% CI 32.2%–41.6%) in Maputo Province, which is a high level for an area in which no RVFV disease activity has been reported during the past 4 decades.

Although the study was designed to determine the overall prevalence in the province, our data also provided an indication of the distribution of RVFV at a district level. Maputo Province is subdivided into 7 districts, and samples from 6 of these districts were available for analysis: Boane (n = 28), Magude (n = 34), Manhiça (n = 65), Marracuene (n = 82), Matutuine (n = 131), and Moamba (n = 64). The livestock populations in Magude, Manhiça, Matutuine, and Moamba Districts range in size from 20,000 to 70,000 animals, and Boane and Marracuene Districts have smaller populations of 6,000 and 9,000, respectively.

We found the highest seroprevalence to be 61.5% (95% CI 49.7%–73.4%) in Manhiça District and 62.2% (95% CI 51.7%–72.7%) in Marracuene District. Some of the animals affected by RVFV during outbreaks in 1969 were raised on farms near or in these 2 regions (*1*). Our data indicate that the RVFV activity is still high in those districts, possibly because breeding of mosquito vectors is promoted by environmental factors, including irrigation ditches on sugar cane farms, extensive flood plains, wetlands, and ponds (*5*).

South Africa keeps continuous records of RVFV outbreaks, and several outbreaks in cattle were reported in KwaZulu-Natal and Mpumalanga Provinces during 2008–2010 (*6*). Matutuine, the southernmost district in Maputo Province, shares a border with KwaZulu-Natal Province, whereas Magude and Moamba Districts border Mpumalanga Province. Accordingly, the high seropositivity rates of 19.8% (95% CI 13.0%–26.7%), 26.5% (95% CI 11.6%–41.3%), and 29.7.% (95% CI 18.5%–40.9%) in Matutuine, Magude, and Moamba Districts, respectively, are not remarkable. In comparison, a seroprevalence of 14.3% (95% CI 1.3%–27.2%) was noted in Boane District, which has a smaller livestock population.

Because of long-term persistence of IgG against RVFV, the actual time of infection could not be determined from the data obtained in our study. However, information was available regarding the age of the cattle in Moamba District. From this information, we deduced that the most recent RVFV infections in this district occurred at some point during 2009–2010.

Our results strongly suggest that RVFV is widely distributed among bovids in Maputo Province, although the modality of this circulation is unknown. RVFV infection can remain undetected in adult livestock but can cause abortions in pregnant animals and neonatal death in small ruminants (*7*), which has major economic consequences. Transmission to humans is common during epizootics, and the proximity to and density of cattle in an area have been shown to be major factors for RVFV seroconversion in human populations (*8*). Human infections are often manifested as a febrile illness and can easily be mistaken for other diseases. Consequently, unidentified or underdiagnosed RVFV infections among livestock in Maputo Province warrant further research, and implementation of surveillance and livestock vaccination programs in the studied area should be discussed.
